# The Use of Machine Learning to Predict Late Arrivals at the Adult Outpatient Department

**DOI:** 10.7759/cureus.39886

**Published:** 2023-06-02

**Authors:** Mohammed D Aldhoayan, Rami M Alobaidi

**Affiliations:** 1 Health Affairs, King Abdulaziz Medical City Riyadh, Riyadh, SAU; 2 Health Informatics, King Saud Bin Abdulaziz University for Health Sciences, Riyadh, SAU; 3 Information Technology, King Abdulaziz Medical City Riyadh, Riyadh, SAU

**Keywords:** analysis, outpatient, data analytics, machine learning, late arrival

## Abstract

Introduction: Patient unpunctuality leads to delays in the delivery of care and increased waiting times, resulting in crowdedness. Late arrivals for adult outpatient appointments are a challenge for healthcare, contributing to negative effects on the efficiency of health services as well as wasted time, budget, and resources. This study aims to identify factors and characteristics associated with tardy arrivals at adult outpatient appointments using machine learning and artificial intelligence. The goal is to create a predictive model using machine learning models capable of predicting adult patients arriving late to their appointments. This would support effective and accurate decision-making in scheduling systems, leading to better utilization and optimization of healthcare resources.

Methods: A retrospective cohort review of adult outpatient appointments between January 1, 2019, and December 31, 2019, was undertaken at a tertiary hospital in Riyadh. Four machine learning models were used to identify the best prediction model that could predict late-arriving patients based on multiple factors.

Results: A total of 1,089,943 appointments for 342,974 patients were conducted. There were 128,121 visits (11.7%) categorized as late arrivals. The best prediction model was Random Forest, with a high accuracy of 94.88%, a recall of 99.72%, and a precision of 90.92%. The other models showed different results, such as XGBoost with an accuracy of 68.13%, Logistic Regression with an accuracy of 56.23%, and GBoosting with an accuracy of 68.24%.

Conclusion: This paper aims to identify the factors associated with late-arriving patients and improve resource utilization and care delivery. Despite the overall good performance of the machine learning models developed in this study, not all variables and factors included contribute significantly to the algorithms' performance. Considering additional variables could improve machine learning performance outcomes, further enhancing the practical application of the predictive model in healthcare settings.

## Introduction

Healthcare organizations consistently allocate resources and time to develop efficient outpatient scheduling systems to optimize resource utilization [1]. The complexity of appointment policy design increases when patients arrive earlier or later than their scheduled appointments [2]. Patient non-adherence to appointment times, encompassing early and late arrivals, poses challenges for patients, physicians, and healthcare personnel. Such deviations in punctuality can lead to delays in care delivery, extended waiting periods, and overcrowding [1].

Inefficiencies in appointment systems and patient punctuality issues negatively impact caregiver productivity and access to care, contributing to annual expenses exceeding $150 billion in the U.S. healthcare system [3]. A 2009 report from the Institute of Medicine estimated that $750 billion of unnecessary health spending occurred in the U.S., with $130 billion attributed to inefficient care delivery. This scenario presents an opportunity for healthcare organizations to enhance the efficiency and quality of their services [4]. While waiting times have been extensively examined, the issue of patient punctuality has not been adequately addressed, potentially leading to suboptimal outcomes [5]. Late patient arrivals remain a prevalent challenge for healthcare organizations [6]. Srinivas reported varying arrival rates across specialties, with around 10% in pediatrics, 22% in urology, and 38% in cardiology [6].

Despite implementing operational strategies such as prioritizing on-time arrivals, offering alternative rescheduling policies, and utilizing automated text message reminders, clinics continue to experience tardy arrivals. Glowacka et al. identified the most common approach for addressing late arrivals as prioritizing them at the top of the patient queue for service [7]. Conversely, early arrivals are managed according to each clinic's specific policies, such as "first in, first out" or adhering strictly to the scheduling system [8]. Non-attendance and late arrivals directly impact healthcare systems, causing financial burdens and clinical inefficiencies. Faiz & Kristoffersen collected variables including age, gender, arrival time, appointment time, and weather for their study [8].

The study found a non-attendance rate of 12.0% (54/449) for new referrals and 8.2% (74/904) for follow-ups. Among the 1,225 attending patients, 63 (5.1%) arrived late. The findings indicated that older patients tended to be more punctual than their younger counterparts, with significant associations between more senior age, early arrival time, and lower non-attendance rates. Srinivas suggested incorporating factors contributing to late arrivals into scheduling system designs [6]. This could involve overlapping appointment times for patients who consistently arrive on time with those anticipated to be late, enabling more effective resource utilization. Healthcare organizations should determine optimal overlapping decisions through further analysis and experimentation.

Considering the challenges above and potential areas for improvement, machine learning (ML) offers a sophisticated and flexible algorithmic solution. Machine learning has recently gained traction in medical fields, demonstrating the ability to outperform human processing capabilities in certain tasks [9,10]. In this study, we propose developing models to predict late arrivals effectively, incorporating factors such as patient gender, no-show history, age, lead time, visit type, and visit time for a comprehensive prediction.

Our objective is to employ machine learning techniques to construct a predictive model for adult late arrival rates at King Abdulaziz Medical City, a tertiary hospital in Riyadh, Saudi Arabia. This predictive model will exhibit high interpretability, enabling effective and accurate decision-making regarding adult late arrivals. Consequently, this approach will facilitate the identification and detection of underutilized adult outpatient appointments, thereby improving the overall efficiency of the healthcare system. To the best of our knowledge, ML has not been used to predict adult late arrival rates.

## Materials and methods

Data collection and preprocessing

Data for this study were obtained from the Electronic Medical Record (EMR) system, spanning from January 1, 2019, to December 31, 2019. The dataset encompassed masked (de-identified) patient demographics (patient information, patient ID, age, and gender), weather information (wind type, wind speed, air temperature, visibility distance, and temperature dew points), and patient visit information (reservation time, arrival time, medical department ID, and appointment details).

A new attribute for age intervals was derived from the age data to examine the relationship between age and arrival status. The intervals included three categories: ages 18 to 35, ages 36 to 50, and ages above 50. This categorization has been chosen based on the hypothesized different groups of patients, according to the expertise involved in this study. A similar approach was applied to time data, creating a new attribute for time intervals (07:00-09:00, 09:00-12:00, 12:00-15:00, 15:00-17:00, and beyond working hours) to investigate potential associations between rush hours and arrival status. Hourly climate data, including wind type, wind speed rate, visibility distance, and temperature, were sourced from the King Abdullah Petroleum Studies and Research Center (KAPSARC) portal [11]. Subsequently, the nearest hourly climate data were mapped to actual check-in times to explore the relationship between weather conditions and arrival status. The outcome variable, arrival status, was classified as either on-time or late arrival, determined using EMR appointment timestamp data. A patient was considered late if the difference between the actual check-in and scheduled visit time exceeded 15 minutes. The independent variables used to predict late arrivals were divided into two categories: patient demographics (age and gender) and appointment characteristics (day of the visit, weather, medical department, and scheduled visit time). Canceled appointments, no-shows, walk-ins, and pre-operation consultation appointments were excluded. Booked and checked-in appointment data were analyzed to develop and validate a machine learning algorithm capable of predicting late-arriving patients.

Handling missing variables

During data preprocessing, duplicate records in the dataset were removed, as were records containing null values. The preprocessing steps also involved oversampling to balance the dataset using the synthetic minority oversampling technique, ensuring equal representation of classes in the outcome variable. Additionally, conversions between categorical and numerical data types were performed as needed.

Statistical analysis and machine learning

To achieve the study's objective, predictive models were employed to forecast late-arriving patients using the aforementioned data elements. Given the binary nature of the arrival status outcome (either on-time or late), four machine learning algorithms were trained and validated for the predictive data task: Random Forest (RF), Gradient Boosting (GB), Logistic Regression (LR), and Extreme Gradient Boosting (XGBoost). Multiple hypertunings were conducted on each model, but all failed to produce better results than the default settings from the Scikit-learn library in Python version 3.9 [12]. These models were chosen based on the dichotomous nature of the outcome and have been shown to be the best-performing models based on the past experience of the data analysts involved in this study. To evaluate the models, the data were split in a 70:30 ratio. Accuracy, precision, recall, and F-measure were metrics for comparing model performance and selecting the most appropriate model.

## Results

A total of 1,089,943 appointment visits for 342,974 patients were included, with females representing 61.73% of the sample. There were 128,121 late-arriving appointments and 1,135,008 on-time visits, with late arrivals accounting for 10.14% of all outpatient visits. A summary of dataset characteristics is shown in Table 1.

**Table 1:** Descriptive characteristics of the dataset

Features	Late (%)	On-time (%)	Total (N)
Gender			
Male	45,527 (9.42%)	437,913 (90.58%)	483,440
Female	82,594 (10.59%)	697,095 (89.41%)	779,689
Time group (hours)			
07:00-09:00	31,001 (20.01%)	123,932 (79.99%)	154,933
09:00-12:00	50,545 (10.32%)	439,073 (89.68%)	489,618
12:00-15:00	33,719 (8.90%)	345,439 (91.10%)	379,158
15:00-17:00	4,424 (3.30%)	129,363 (96.70%)	133,787
Beyond working hours	8,432 (7.98%)	97,201 (92.02%)	105,633
Age group (years)			
18-25	18,853 (10.86%)	154,729 (89.14%)	173,582
26-35	26,486 (9.70%)	246,308 (90.30%)	272,794
36-50	31,558 (9.29%)	308,273 (90.71%)	339,831
51-65	31,747 (10.48%)	271,154 (89.52%)	302,901
66-85	11,408 (11.27%)	89,830 (88.73%)	101,238
> 85	8,069 (11.09%)	64,714 (88.91%)	72,783
Day			
Monday	26,857 (9.99%)	242,040 (90.01%)	268,897
Tuesday	27,901 (10.33%)	242,381 (89.67%)	270,282
Wednesday	26,693 (10.30%)	232,430 (89.70%)	259,123
Thursday	18,800 (9.78%)	173,150 (90.22%)	191,950
Friday	551 (9.62%)	5,174 (90.38%)	5,725
Saturday	1,285 (12.71%)	8,822 (87.29%)	10,107
Sunday	26,034 (10.13%)	231,011 (89.87%)	257,045

The age interval and arrival status distribution of outpatient visits show that patients older than 65 years old were most likely to arrive late for their appointments compared to patients less than 65 years old. The gender distribution of outpatient visits shows that male patients (9.42%) were less likely to arrive late for their appointments than female patients (10.59%). The time interval distribution of outpatient visits shows that late arrivals for clinics before noon were higher than after. The day distribution of outpatient visits shows that Saturday had a higher rate of late arrivals than other days.

The feature importance results from testing the Random Forest (RF) model show that the top three important predictors are age, medical department, and air temperature. Other factors, like wind type and visibility distance, have less influence on arrival status. Predictors like the year, scheduled visit time, and actual check-in were removed as they do not add value to the model.

Performance of the models and predictor

The best prediction model was the Random Forest, with a high accuracy of 98.88%, a high recall of 99.72%, and a high precision of 90.92%. The other models showed different results: XGBoost had an accuracy of 68.13%, Logistic Regression had an accuracy of 56.23%, and gradient boosting had an accuracy of 68.24%. Table 2 shows the performance measures of the four models used in this study.

**Table 2:** Performance of machine learning models

Model Name	Accuracy	AUC	Recall	Precision	F1-Score
Random Forest (RF)	98.88%	.99	99.72%	90.92%	95.12%
Logistic Regression (LR)	56.23%	.59	59.24%	55.88%	57.51%
Extreme Gradient Boosting (XGBoost)	68.13%	.75	73.40%	66.40%	69.72%
Gradient Boosting (GB)	68.24%	.76	73.43%	66.53%	69.81%

Logistic Regression achieved the best training time performance using a ratio of 70:30. On the other hand, the Random Forest was 10 times slower than gradient boosting, although it achieved optimal accuracy, the area under the curve (AUC), recall, precision, and F1-score results. Training and test time are key criteria, especially for large datasets. As part of the machine learning (ML) model evaluation criteria, training and test time (in seconds) were measured for each algorithm, as shown in Table 3.

**Table 3:** Training and testing time values for machine learning models

Algorithm name	Training time (in seconds)	Testing time (in seconds)
Random Forest	300	40
XGBoost	220	35
Logistic Regression	30	15
Gradient Boosting Machine	350	45

Furthermore, the feature importance for the predictor variables has been analyzed, and the results are shown in Table 4.

**Table 4:** Feature importance based on the Random Forest model

Feature	Importance
Air temperature	0.17
Gender	0.14
Wind speed rate	0.13
Visibility distance	0.07
Age	0.03
Hour	0.01
Day	0.01

## Discussion

Several studies have focused on predicting patient punctuality and no-shows [13,14]. Another category of studies examined the clinical risks of patient punctuality [15,16]. In this study, we tried to identify the factors associated with late-arriving patients using data available in the EMR. Comparing the findings and results of this study with other study findings performed by Srinivas [6], Wainer [17], and Zhou et al. [18], RF and GB algorithms are found to have better predictive performance measures for the dataset examined in this study. However, it was found that the RF algorithm provided the highest accuracy, recall, and precision in our study.

In contrast to other studies that have focused on identifying factors associated with patient punctuality for specific clinics Srinivas [6], or studies that focused on the association between specific factors and outpatient clinic arrival time (Faiz and Kristoffersen [8]), this study focused on studying and identifying the factors associated with late arriving patients based on patient demographic information, appointment characteristics, as well as weather conditions.

Consistent with the findings of Srinivas [6] and Faiz and Kristoffersen [8], in this study, age has an impact on patient unpunctuality. Also of note, weather conditions have a great influence on arrival status. This is clear from the air temperature's importance in the feature importance analysis. In general, the analysis of this study indicated that the information available in the EMR is fair enough to predict late arrivals with acceptable levels of accuracy using ML models. The ML algorithm, i.e., RF, is found to have significantly better predictive performance for the dataset considered in this research.

As Wiens and Shenoy [19] indicated, an ML algorithm achieving an acceptable level of performance measures would add value to healthcare organizations. This study's findings demonstrate the ML model's ability to predict tardy arrivals accurately in advance, enabling healthcare organizations to manage and mitigate patient unpunctuality effectively and efficiently for better decision-making. In reality, clinics deal with both scenarios: no-shows and late arrivals. Therefore, it would be beneficial for care providers to design ML models to predict the status of arrival, considering on-time, no-show, and late arrival, in order to have better visibility and make informed decisions. Having such a model would help caregivers better design their appointment and scheduling systems. As suggested by Srinivas [6], overlapping the appointment time of a patient predicted to arrive on time with another patient who is probably going to arrive late could lead to compensation for the delayed interval.

It is worth mentioning that, similar to other studies, this study also has some limitations that need to be addressed. There are some missing variables that were not collected, such as the distance between the patient’s home and hospital, whether the patient is coming to the hospital through public transportation if they are traveling from another city, and information about the availability of clinical staff. Besides, there were no interviews conducted with late-arriving patients to record the reasons for not coming on time. Future studies could include these data to better explain late arrivals at outpatient visits.

## Conclusions

Although predicting the arrival status of patients is not a straightforward task due to a variety of factors that could affect patient punctuality, this study provides valuable insights into the predictors affecting patients' arrival on time for a scheduled visit. This study has demonstrated that machine-learning models' are showing promising performance measures in detecting late arrivals. The results show that weather, gender, and age have the potential to explain late arrivals. However, although the model's accuracy is high, adding more attributes related to factors like the distance between the hospital and the patient's residence, traffic data at the visit, and whether the patient is traveling could result in even better accuracy. Future studies should consider including datasets from multiple hospitals to increase the generalizability and robustness of the results. These key and important factors will likely increase the accuracy and performance of the model, further enhancing its practical application in healthcare settings for improved patient scheduling and resource management.

## References

[1] Zhu H, Chen Y, Leung E, Liu X (2018). Outpatient appointment scheduling with unpunctual patients. Int J Prod Res.

[2] Klassen KJ, Yoogalingam R (2014). Strategies for appointment policy design with patient unpunctuality. Decis Sci.

[3] Srinivas S, Ravindran AR (2018). Optimizing outpatient appointment system using machine learning algorithms and scheduling rules: a prescriptive analytics framework. Expert Syst App.

[4] Yu S, Kulkarni VG, Deshpande V (2020). Appointment scheduling for a health care facility with series patients. Prod Oper Manag.

[5] Gorodeski EZ, Joyce E, Gandesbery BT (2017). Discordance between 'actual' and 'scheduled' check-in times at a heart failure clinic. PLoS One.

[6] Srinivas S (2020). A machine learning-based approach for predicting patient punctuality in ambulatory care centers. Int J Environ Res Public Health.

[7] Glowacka KJ, May JH, Goffman RM (2017). On prioritizing on-time arrivals in an outpatient clinic. IISE Transactions on Healthcare Systems Engineering.

[8] Faiz KW, Kristoffersen ES (2018). Association between age and outpatient clinic arrival time: myth or reality?. BMC Health Serv Res.

[9] Tseng YJ, Huang CE, Wen CN (2019). Predicting breast cancer metastasis by using serum biomarkers and clinicopathological data with machine learning technologies. Int J Med Inform.

[10] Curtis C, Liu C, Bollerman TJ, Pianykh OS (2018). Machine learning for predicting patient wait times and appointment delays. J Am Coll Radiol.

[11] (2022). Data & tools: access our open data, research tools and policy simulators. https://www.kapsarc.org/research/data-tools/.

[12] Pedregosa F, Varoquaux G, Gramfort A (2011). Scikit-learn: machine learning in Python. J Mach Learn Res.

[13] Robaina JA, Bastrom TP, Richardson AC, Edmonds EW (2020). Predicting no-shows in paediatric orthopaedic clinics. BMJ Health Care Inform.

[14] Daghistani T, AlGhamdi H, Alshammari R, AlHazme RH (2020). Predictors of outpatients’ no-show: big data analytics using apache spark. J Big Data.

[15] Lopez-Martinez F, Schwarcz A, Núñez-Valdez ER, Garcia-Diaz V (2018). Machine learning classification analysis for a hypertensive population as a function of several risk factors. Expert Syst Appl.

[16] Weng SF, Reps J, Kai J, Garibaldi JM, Qureshi N (2017). Can machine-learning improve cardiovascular risk prediction using routine clinical data?. PLoS One.

[17] Wainer J (2016). Comparison of 14 different families of classification algorithms on 115 binary datasets. arXiv preprint arXiv:160600930.

[18] Zhou J, Li X, Mitri HS (2016). Classification of rockburst in underground projects: comparison of ten supervised learning methods. J Comput Civ Eng.

[19] Wiens J, Shenoy ES (2018). Machine learning for healthcare: on the verge of a major shift in healthcare epidemiology. Clin Infect Dis.

